# Supplementary material for: Content-rich biological network constructed by mining PubMed abstracts
Source: BMC Bioinformatics. 2004 Oct 8;5:147. doi: 10.1186/1471-2105-5-147 (PMC528731; doi:10.1186/1471-2105-5-147)
Supplement: Additional File 2 — The original results of the above study (non-essential files are deleted to keep the file size under the limit set by BMC bioinformatics). [file 1471-2105-5-147-S2.bz2 › chilibotAdditionalFile2/dip10/463ID11259614E10490/html/CXCL11_CXCR3.html]

 


 **CXCL11** and **CXCR3** 
  
Found 66 abstracts in PubMed, retrieved 10.  
 

 What does Google say? 
 PDF only 
| .edu only 

---

**Interactive relationship** (e.g. stimulation, inhibition, etc)

**Inhibitory relationship**- An Alternatively Spliced Variant of  **CXCR3**  Mediates the Inhibition of Endothelial Cell Growth Induced by IP 10  [ **CXCR3** ] , Mig, and I TAC  [ **CXCL11** ] , and Acts as Functional Receptor for Platelet Factor 4.  Ref: 12782716 J Exp Med, 2003
- We alsodemonstrate that the  **CXCR3**  ligands inhibit CCR3 mediated functional responses of both human eosinophils and CCR3 transfectants induced by all three eotaxins, with  **CXCL11**  being the most efficacious antagonist.  Ref: 12884299 Eur J Immunol, 2003
**Neutral relationship**- Human microvascular endothelial cell line 1 HMEC 1, transfected with either the known  **CXCR3**  renamed  **CXCR3**  A or  **CXCR3**  B, bound CXCL9, CXCL10, and  **CXCL11** .  Ref: 12782716 J Exp Med, 2003
- The chemokine receptor  **CXCR3**  is predominantly expressed on T lymphocytes, and its agonists CXCL9, CXCL10 and  **CXCL11**  are IFN gamma inducible chemokines that promote Th1 responses.  Ref: 12884299 Eur J Immunol, 2003

**Non-interactive relationship** (e.g. studied together, co-existance, homology, etc.)

- Immunohistochemical localization confirmed the presence of  **CXCR3**  mononuclear cells within lesions and the presence of the ligand, ITAC  **CXCL11** , on the surface of endothelial cells associated with TCAD.  Ref: 12695288 Circulation, 2003
- Interferon gamma inducible chemokines CXCL9 Mig, CXCL10 IP 10  [ **CXCR3** ] ,  **CXCL11**  I TAC  [ **CXCL11** ] , strongly associated to Th1 mediated immune responses, belong to the CXC sub family.  Ref: 12739748 J Endocrinol Invest, 2003
- Lesional keratinocytes also overexpressed the ligands of  **CXCR3** , namely, MIG CXCL9, CXCL10, and I TAC  [ **CXCL11** ]   **CXCL11** .  Ref: 12819030 Am J Pathol, 2003
- Remarkably, primary cultures of human microvascular endothelial cells, whose growth is inhibited by CXCL9, CXCL10,  **CXCL11** , and CXCL4, expressed  **CXCR3**  B, but not  **CXCR3**  A.  Ref: 12782716 J Exp Med, 2003
- In this study, we first demonstrate that in COS 7 cells heterologously expressing  **CXCR3** ,  **CXCL11**  is a potent activator of the pertussis toxin sensitive PTX p44 p42 MAPK and Akt PI 3 kinase pathways.  Ref: 12750173 Blood< MedlineTA>Blood, 2003
- Real time, quantitative reverse transcriptase polymerase chain reaction analysis demonstrates that the  **CXCR3**  ligands, interferon IFN inducible protein 10 CXCL10 and IFN inducible T cell alpha chemoattractant  **CXCL11** , are among the many chemokines induced in the adjuvant treated peritoneum.  Ref: 12773510 J Leukoc Biol, 2003
- This review discusses chemokine responses in relation to HSV infection of the cornea emphasizing the role of  **CXCR3**  chemokine signaling by the IFN gamma inducible ligands MIG, IP10  [ **CXCR3** ]  and I TAC  [ **CXCL11** ]  and includes discussion of their potential role in immunopathology in the nervous system.  Ref: 12815542 Curr Eye Res, 2003
- Potential molecular differences between early and late rejection grade > =2 episodes were examined by testing chemokine and receptor gene expression.METHODS Expression of inducible protein IP 10  [ **CXCR3** ] , monokine induced by IFN gamma Mig, interferon inducible T cell alpha chemoattractant  [ **CXCL11** ]  I TAC  [ **CXCL11** ] , regulated on activation normal T cell expressed and secreted RANTES, and their receptors  **CXCR3**  and CCR5 was tested in 60 endomyocardial biopsies from 24 patients using quantitative Taqman real time polymerase chain reaction PCR.  Ref: 12829909 Transplantation, 2003
- The chemokines CXCL9 Mig, CXCL10 IP 10  [ **CXCR3** ] , and  **CXCL11**  I TAC  [ **CXCL11** ]  regulate lymphocyte chemotaxis, mediate vascular pericyte proliferation, and act as angiostatic agents, thus inhibiting tumor growth.  Ref: 12782716 J Exp Med, 2003
- Moreover, cytokines induced IL 15, IP 10  [ **CXCR3** ] , Mig, I TAC  [ **CXCL11** ]  and MIP 3alpha protein accumulation in culture medium from human islets.  Ref: 12627325 Diabetologia, 2003
- CCR3 functional responses are regulated by both  **CXCR3**  and its ligands CXCL9, CXCL10 and  **CXCL11** .  Ref: 12884299 Eur J Immunol, 2003
- Immunohistochemical localization of the  **CXCR3**  chemokine ligand ITAC  **CXCL11**  on the endothelial surface of TCAD lesions with underlying infiltration of inflammatory mononuclear cells expressing  **CXCR3**  suggests a causative role for this chemokine in the development of TCAD.  Ref: 12695288 Circulation, 2003
- CONCLUSIONS Elevated peripheral blood levels of the  **CXCR3**  chemokine ITAC  **CXCL11**  are associated with severe TCAD and may serve as a marker for patients at increased risk for the development of this disease.  Ref: 12695288 Circulation, 2003
